# Internet Use, Risk Awareness, and Demographic Characteristics Associated With Engagement in Preventive Behaviors and Testing: Cross-Sectional Survey on COVID-19 in the United States

**DOI:** 10.2196/19782

**Published:** 2020-06-16

**Authors:** Siyue Li, Bo Feng, Wang Liao, Wenjing Pan

**Affiliations:** 1 College of Media and International Culture Zhejiang University Hangzhou China; 2 Department of Communication University of California at Davis Davis, CA United States; 3 School of Journalism and Communication Renmin University of China Beijing China

**Keywords:** COVID-19, coronavirus, preventive behaviors, testing, online health information, risk awareness

## Abstract

**Background:**

During the coronavirus disease (COVID-19) pandemic, engagement in preventive behaviors and getting tested for the virus play a crucial role in protecting people from contracting the new coronavirus.

**Objective:**

This study aims to examine how internet use, risk awareness, and demographic characteristics are associated with engagement in preventative behaviors and testing during the COVID-19 pandemic in the United States.

**Methods:**

A cross-sectional survey was conducted on Amazon Mechanical Turk from April 10, 2020, to April 14, 2020. Participants’ internet use (in terms of the extent of receiving information pertaining to COVID-19), risk awareness (whether any immediate family members, close friends or relatives, or people in local communities tested positive for COVID-19), demographics (sex, age, ethnicity, income, education level, marital status, and employment status), as well as their engagement in preventative behaviors and testing were assessed.

**Results:**

Our data included 979 valid responses from the United States. Participants who received more COVID-19–related health information online reported more frequent effort to engage in all types of preventive behaviors: wearing a facemask in public (odds ratio [OR] 1.55, 95% CI 1.34-1.79, *P*<.001), washing hands (OR 1.58, 95% CI 1.35-1.85, *P*<.001), covering nose and mouth when sneezing and coughing (OR 1.78, 95% CI 1.52-2.10, *P*<.001), keeping social distance with others (OR 1.41, 95% CI 1.21-1.65, *P*<.001), staying home (OR 1.40, 95% CI 1.20-1.62, *P*<.001), avoiding using public transportation (OR 1.57, 95% CI 1.32-1.88, *P*<.001), and cleaning frequently used surfaces (OR 1.55, 95% CI 1.34-1.79, *P*<.001). Compared with participants who did not have positive cases in their social circles, those who had immediate family members (OR 1.48, 95% CI 8.28-26.44, *P*<.001) or close friends and relatives (OR 2.52, 95% CI 1.58-4.03, *P*<.001) who tested positive were more likely to get tested. Participants’ sex, age, ethnicity, marital status, and employment status were also associated with preventive behaviors and testing.

**Conclusions:**

Our findings revealed that the extent of receiving COVID-19–related information online, risk awareness, and demographic characteristics including sex, ethnicity, age, marital status, and employment status are key factors associated with US residents’ engagement in various preventive behaviors and testing for COVID-19.

## Introduction

Since its initial outbreak in late December 2019, the coronavirus disease (COVID-19) pandemic has caused and is continuing to cause a severe, large-scale impact on individuals and societies across the world, including the United States [1,2]. As of May 29, 2020, there were over 1.7 million confirmed COVID-19 cases and more than 100,000 deaths in the United States [3]. In the face of a rapidly growing pandemic such as COVID-19, prompt and up-to-date assessment of the public’s behavioral responses to the pandemic is critical if the findings are to be informative to public health policies and responses at local, regional, and national levels [4]. Compared with previous pandemics, the COVID-19 pandemic poses unprecedented challenges to public health responses due in part to its unique epidemiological characteristics. For example, the incubation period of COVID-19 can be as long as 24 days, and studies have found that a significant proportion of individuals infected with COVID-19 were asymptomatic but highly contagious, thus, posing enormous challenges for containing the spread of COVID-19 [5,6]. For the time being, there are no vaccines or antiviral medicine to treat or prevent this novel coronavirus [7]. Given all these factors, it is imperative for the public to actively engage in preventive behaviors and testing for the virus [8]. Correspondingly, research that identifies potential predictors of engagement in preventive behaviors and testing will generate urgently needed insights into social responses to the pandemic and inform targeted interventions to promote preventive behaviors and testing.

At the time of writing, we are aware of only one study that examined engagement in preventive measures (eg, avoiding in-person social interactions, staying home, washing hands) during the early stage (early March 2020) of the pandemic in the United States [9]. Although another study examined the use of masks as a behavioral response during the pandemic, this behavior was not examined as a preventive measure but instead as a behavioral response against the Centers for Disease Control and Prevention (CDC) and National Institute of Health (NIH) recommendations [10]. To our knowledge, no research has examined testing behaviors during the pandemic. Large-scale testing, followed by contact tracing and isolation of those with positive test results, is an essential measure for preventing a large fraction of possible transmission chains [8,11,12].

As a new infectious disease, COVID-19 has triggered a massive spike in uncertainty among the public. To learn more about the disease and to better cope with the pandemic, people are motivated to acquire relevant information through various sources. The internet has become a particularly important source of health information [13]. Recent research shows that people rely heavily on the internet to search for relevant COVID-19 health information [14]. Besides acquiring information through active search, people are also incidentally exposed to health information online [15]. Health information received online not only fills an information gap but also influences people’s health decision making [16,17]. Besides the internet, personal experiences serve as a prominent means to acquire information. Because the virus is primarily transmitted through personal contacts, awareness of infection in one’s social surroundings, including immediate family, friends and relatives, and local communities, are likely to affect people’s risk perceptions and their engagement in preventive behaviors and testing. Past research has also shown that different demographic characteristics tend to be correlated with preventive behaviors during a pandemic [18]. A newly published article noted that demographic characteristics such as age and sex were associated with COVID-19 fatality rates [19], suggesting a necessary role of demographic factors in the investigation of behavioral responses during the COVID-19 pandemic. In this study, we examine internet use, risk awareness, and demographic characteristics associated with preventive behaviors and testing during the COVID-19 pandemic in the United States. Internet use is primarily examined via the amount of COVID-19–related information one has received online, including information received from both active search and passive exposure. Risk awareness is conceptualized as the extent that people have knowledge of infections in their social surroundings. Major demographic characteristics including sex, age, ethnicity, income, education, marital status, and employment status were also examined in this study.

## Methods

### Sampling Participants

This study received ethical approval from the corresponding author’s university. Participants for the study were recruited from Amazon Mechanical Turk (MTurk), an online crowdsourcing labor marketplace operated by Amazon. There is evidence showing that MTurk samples provide data equivalent in quality to the data generated from alternative samples [20]. The survey was constructed and administered using Qualtrics (version 12; Qualtrics International Inc). Qualtrics records individual responses to the survey but not the MTurk account information, so participants remain anonymous. Each participant received US $0.75 for their participation.

### Data Collection

Data collection started on April 10, 2020, and was completed on April 14, 2020. Upon consent, participants were instructed to complete a survey asking about their perceptions and behaviors related to the COVID-19 pandemic. Specifically, each participant was asked about whether or not they got tested for the virus, their engagement with different types of preventive behaviors, the extent to which they received information related to COVID-19, the amount of time they spent on the internet on a daily basis, and risk awareness regarding others’ COVID-19–related health status (see Textbox 1). For engagement in preventative behaviors against COVID-19, items were measured on a 5-point scale (1=never, 2=sometimes, 3=about half the time, 4=most of the time, 5=always) and were prefaced with the question “Over the past month, how often have you engaged in the following practices to minimize the risk of contracting the coronavirus (COVID-19)?” For receiving COVID-19–related health information online, items were measured on a 5-point scale (1=didn’t receive at all, 2=received rarely, 3=received occasionally, 4=received regularly, 5=received a great deal) and were prefaced with the question “Over the past month, to what extent have you received the following types of informational support online?” Extent of receiving COVID-19–related information was calculated based on the average of the four items (Cronbach alpha=.84). Based on the recommendations from the World Health Organization and the CDC in the United States [7,21], seven types of preventive behaviors were examined in this study: wearing a facemask, washing hands, covering nose and mouth when sneezing and coughing, social distancing, staying home, avoiding public transportation, and cleaning frequently touched surfaces. Risk awareness of others’ COVID-19–related health status was assessed by asking if the participants were aware of any positive cases in their immediate family, among close friends and relatives, or in local communities. The survey also obtained participants’ basic demographic information including sex, age, ethnicity, income, marital status, educational level, and employment status.

In total, 1080 MTurk workers filled out the online questionnaire. To ensure data quality, we included attention checks in the questionnaire. At three different places in the questionnaire, participants were asked to select a designated answer without giving a specific content question (eg, “Please select ‘Never’ for this question,” “Please select ‘Disagree’ for this question”). Failure to select the designated answer for any of the three questions was considered an indication of random clicking. This resulted in the exclusion of 101 participants. The final data set included 979 participants’ survey responses.

**Measurement of engagement in preventive behaviors and online information reception.**

**Engagement in preventive behaviors against the coronavirus disease (COVID-19)**

- Wear a facemask in public even if I am not sick
- Wash hands regularly for 20 seconds, with soap and water or alcohol-based hand rub
- Cover nose and mouth with a disposable tissue or flexed elbow when coughing or sneezing
- Keep safe social distance with others
- Stay home
- Avoid using public transportation
- Clean and disinfect frequently touched surfaces such as doorknobs, phones, and keyboards daily

**Received COVID-19–related health information online**

- Information regarding the scientific facts (eg, symptoms, causes of the disease) related to the pandemic
- Information regarding how to prevent contracting the virus
- Information regarding the spreading of the virus
- Information regarding the sources and resources to give and receive social support during the pandemic

### Data Analysis

To assess the effects of internet use, risk awareness, and demographic characteristics on engagement in preventive behaviors and testing of COVID-19, we conducted ordinal logistic regression for the 5-level self-reported engagement in preventive behaviors and binomial logistic regression analysis for the binary testing behavior. In both ordinal and binomial logistic regression analyses, participants’ *demographic characteristics* (ie, sex, age, ethnicity, income, education level, marital status, employment status) were entered in step 1 (see Model 1s in Multimedia Appendix 1), *internet use* (time spent on the internet and the extent of receiving COVID-19–related information online) was entered in step 2 (see Model 2s in Multimedia Appendix 1), and *risk awareness* (whether any immediate family members, close friends or relatives, and people in the local community tested positive for COVID-19) was entered in step 3 (see Model 3s in Multimedia Appendix 1). Detailed results are reported in Multimedia Appendix 1. SPSS 26 (IBM Corp) was used to perform the analyses.

Reported in the Results section, the findings pertaining to the effects of each demographic characteristic reflect their independent effects controlling for other demographic characteristics (ie, based on Model 1s in Multimedia Appendix 1). Findings pertaining to the effects of internet use or risk awareness reflect their independent effects controlling for all other predictors (ie, based on Model 3s in the Multimedia Appendix 1).

## Results

### Participant Characteristics

Demographic characteristics of the 979 participants are provided in Table 1.

**Table 1:** Sample characteristics (n=979).

Demographics			Participants
Female, n (%)			466 (47.6)
**Age groups (years), n (%)**			
	18-27		210 (21.5%)
	28-37		391 (39.9%)
	38-47		196 (20.0%)
	48-57		104 (10.6%)
	≥58		78 (8%)
Age (years), mean (SD)			36.94 (11.93)
**Marital status, n (%)**			
	**Single**		**449 (45.9)**
		Single	376 (38.4)
		Widowed	8 (0.8)
		Divorced	54 (5.5)
		Separated	11 (1.1)
	Married/domestic partnership		530 (54.1)
**Employment status, n (%)**			
	**Working**		**793 (81.0)**
		Self-employed	134 (13.7)
		Working full time for wages	568 (58)
		Working part time for wages	91 (9.3)
	**Not working**		**186 (19.0)**
		Out of work	97 (9.9)
		Not able to work or disabled	18 (1.8)
		Retired	25 (2.6)
		Other	46 (4.7)
**Race, n (%)**			
	White		677 (69.2)
	Black or African American		112 (11.4)
	Hispanic or Latino American		46 (4.7)
	Asian or Asian American		96 (9.8)
	Other		48 (4.9)
**Education level, n (%)**			
	Less than high school degree		4 (0.4)
	High school graduate		63 (6.4)
	Some college but no degree		148 (15.1)
	Associate degree in college		104 (10.6)
	Bachelor’s degree in college		447 (45.7)
	Master’s degree		176 (18.0)
	Doctoral degree		21 (2.1)
	Professional degree (JD, MD)		16 (1.6)
Education year^a^, mean (SD)			15.62 (2.15)
**Household income (US $), n (%)**			
	<10,000		45 (4.6)
	10,001-20,000		76 (7.8)
	20,001-40,000		183 (18.7)
	40,001-60,000		222 (22.7)
	60,001-80,000		201 (20.5)
	80,001-100,000		106 (10.8)
	100,001-120,000		64 (6.5)
	>120,000		82 (8.4)

^a^Education year is transformed from education level based on typical years of completion.

### Engagement in Preventive Behaviors

**Figure 1:**
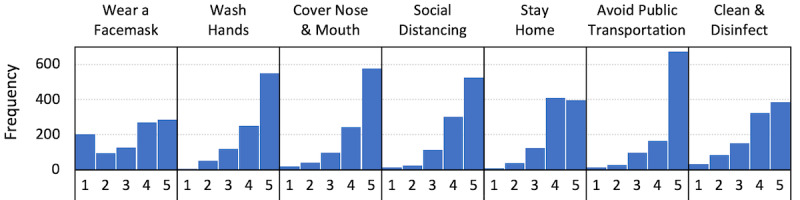
Response distributions of the self-reported preventative behaviors. Responses were coded from 1 (Never) to 5 (Always).

As shown in Figure 1, participants reported less frequent engagement in wearing a facemask in public than other preventive measures (mean 3.35, SD 1.50), and a fair number of participants reported that they never wore a facemask during the pandemic. A large number of participants reported that they frequently washed hands (mean 4.31, SD 0.93), covered nose and mouth when sneezing and coughing (mean 4.35, SD 0.95), kept social distance (mean 4.33, SD 0.87), stayed home (mean 4.17, SD 0.86), avoided using public transportation (mean 4.49, SD 0.89), and cleaned frequently touched surfaces (mean 3.97, SD 1.09) to protect themselves from COVID-19.

#### Internet Use and Engagement in Preventive Behaviors

Our data showed that the amount of COVID-19–related health information received online was positively associated with engagement in all types of preventive behaviors: wearing a facemask in public (odds ratio [OR] 1.55, 95% CI 1.34-1.79, *P*<.001), washing hands (OR 1.58, 95% CI 1.35-1.85, *P*<.001), covering nose and mouth when sneezing and coughing (OR 1.78, 95% CI 1.52-2.10, *P*<.001), keeping social distance with others (OR 1.41, 95% CI 1.21-1.65, *P*<.001), staying home (OR 1.40, 95% CI 1.20-1.62, *P*<.001), avoiding using public transportation (OR 1.57, 95% CI 1.32-1.88, *P*<.001), and cleaning frequently used surfaces (OR 1.55, 95% CI 1.34-1.79, *P*<.001).

#### Risk Awareness and Engagement in Preventive Behaviors

**Figure 2:**
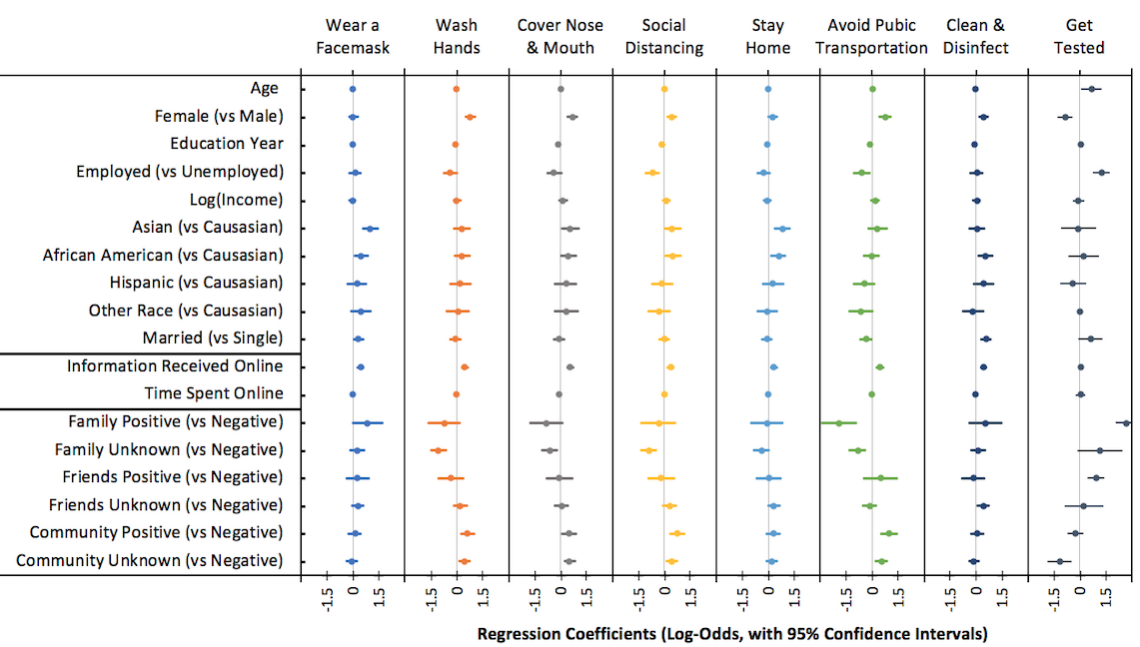
Summaries of effect estimates (standardized) and 95% CIs based on logistic regression models predicting self-reported preventative behaviors (ordered log-odds) and testing behaviors (log-odds).

Our data revealed that awareness of immediate family members’ test results was associated with participants’ engagement in preventive behaviors (see Figure 2 and Model 3s in Multimedia Appendix 1). Specifically, compared with participants who did not have immediate family members with positive test results, those who had immediate family members with positive results less often washed hands (OR 0.35, 95% CI 0.23-0.52, *P*<.001), covered nose and mouth when sneezing and coughing (OR 0.53, 95% CI 0.35-0.80, *P*=.003), kept social distance with others (OR 0.40, 95% CI 0.26-0.61, *P*<.001), and avoided using public transportation (OR 0.42, 95% CI 0.28-0.65, *P*<.001).

Participants reported more frequent effort in cleaning frequently touched surfaces if they had close friends or relatives who tested positive (OR 1.52, 95% CI 1.10-2.10, *P*=.01). In addition, participants who were aware of positive cases in their local communities reported more frequent hand washing (OR 1.54, 95% CI 1.15-2.06, *P*=.004), covering nose and mouth when sneezing and coughing (OR 1.68, 95% CI 1.25-2.26, *P*=.005), keeping social distance (OR 1.51, 95% CI 1.13-2.02, *P*<.001), and avoiding using public transportation (OR 1.71, 95% CI 1.24-2.37, *P*=.001) than those who reported no positive cases in their communities.

#### Demographic Characteristics and Engagement in Preventive Behaviors

**Figure 3:**
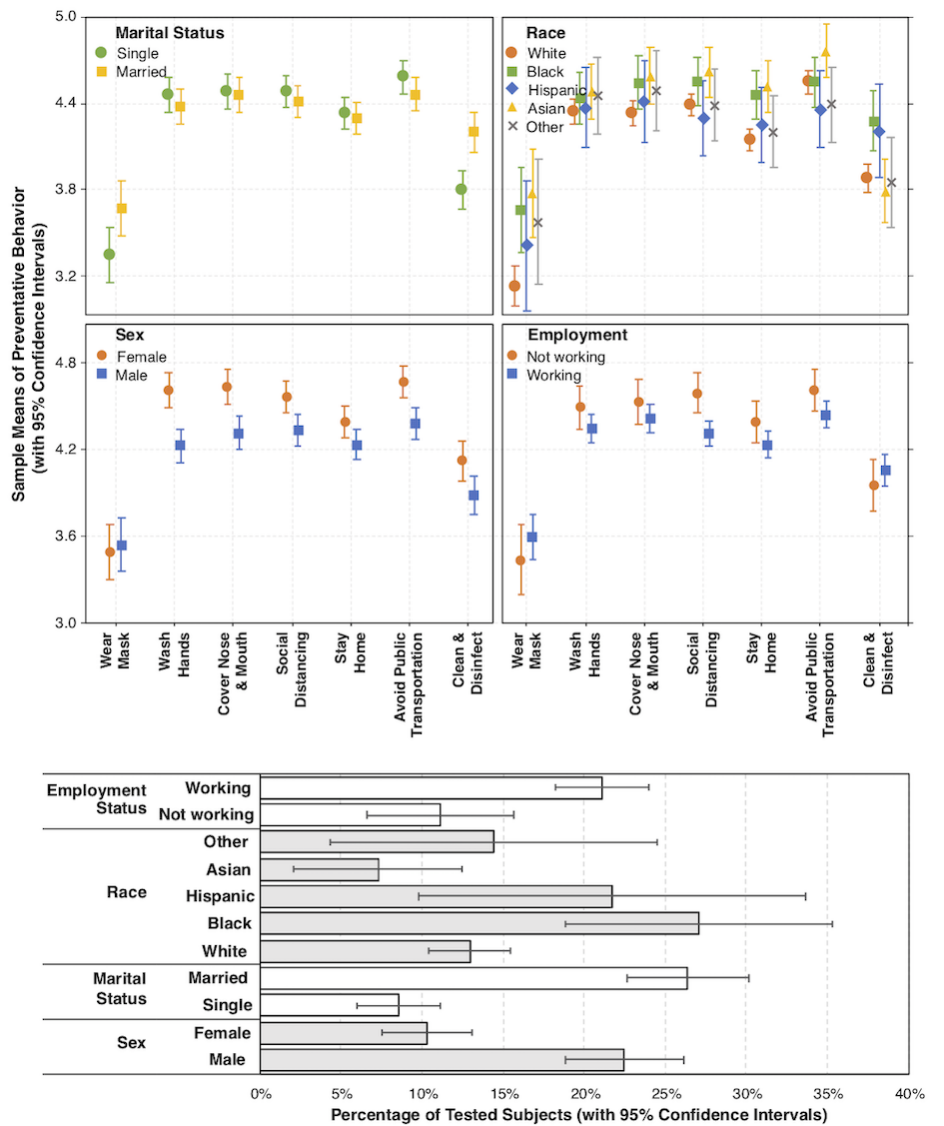
Sample means of self-reported preventative behaviors and proportions of testing (with 95% CIs) across categorical demographic characteristics.

As shown in Figures 2 and 3, several demographic characteristics were found to be associated with engagement in preventive behaviors (also see Model 1s in Multimedia Appendix 1). Compared with males, females more frequently washed their hands (OR 2.39, 95% CI 1.85-3.09, *P*<.001), covered their nose and mouth when sneezing and coughing (OR 2.12, 95% CI 1.63-2.74, *P*<.001), kept social distance with others (OR 1.64, 95% CI 1.28-2.11, *P*<.001), stayed home (OR 1.34, 95% CI 1.05-1.70, *P*=.02), avoided using public transportation (OR 2.30, 95% CI 1.72-3.07, *P*<.001), and cleaned frequently touched surfaces (OR 1.58, 95% CI 1.25-2.00, *P*<.001).

Compared with younger participants, older participants reported more frequent efforts to wash hands (OR 1.01, 95% CI 1.00-1.02, *P*=.04), cover nose and mouth when sneezing and coughing (OR 1.01, 95% CI 1.00-1.03, *P*=.02), keep social distance (OR 1.02, 95% CI 1.01-1.03, *P*=.001), stay home (OR 1.01, 95% CI 1.00-1.02, *P*=.02), and avoid public transportation (OR 1.02, 95% CI 1.01-1.04, *P*<.001) than younger participants.

Ethnic differences were also observed in engagement with preventive behaviors. Compared with whites, African Americans and Asians more frequently wore a facemask in public (OR 1.81, 95% CI 1.26-2.59, *P*<.001; OR 2.47, 95% CI 1.65-3.69, *P*<.001, respectively) and stayed home (OR 1.88, 95% CI 1.28-2.77, *P*=.001; OR 2.23, 95% CI 1.47-3.37, *P*<.001, respectively). In addition, compared with whites, Asians covered their noses and mouths when sneezing and coughing more often (OR 1.78, 95% CI 1.13-2.80, *P*=.01), and kept social distance more often (OR 1.61, 95% CI 1.04-2.48, *P*=.03). African Americans reported more frequent effort in cleaning frequently touched surfaces than whites (OR 2.00, 95% CI 1.36-2.94, *P*<.001).

More educated participants less frequently engaged in the following preventive behaviors: washing hands (OR 0.93, 95% CI 0.87-0.99, *P*=.03), covering nose and mouth when sneezing and coughing (OR 0.87, 95% CI 0.81-0.93, *P*<.001), keeping social distance (OR 0.90, 95% CI 0.84-0.96, *P*=.001), avoiding using public transportation (OR 0.88, 95% CI 0.82-0.95, *P*<.001), and cleaning and disinfecting touched surfaces (OR 1.25, 95% CI 1.04-1.50, *P*=.02).

Participants who are married or have domestic partners more frequently wore a facemask in public (OR 1.43, 95% CI 1.11-1.84, *P*=.01), kept social distance with others (OR 1.64, 95% CI 1.28-2.11, *P*<.001), and cleaned frequently touched surfaces (OR 1.84, 95% CI 1.42-2.38, *P*<.001) than their single counterparts. However, compared to single participants, participants who are married or have domestic partners less frequently avoided public transportation (OR 0.90, 95% CI 0.84-0.96, *P*=.001).

Compared with unemployed participants, employed participants less frequently kept social distance (OR 0.50, 95% CI 0.35-0.72, *P*<.001) or avoided using public transportation (OR 0.56, 95% CI 0.36-0.86, *P*=.01).

Compared to the participants who reported having lower income, the ones reported to have higher income covered their noses and mouths more often (OR 1.25, 95% CI 1.04-1.50, *P*=.02), and avoided public transportation more often (OR 1.29, 95% CI 1.06-1.58, *P*=.01).

### Testing Behavior

Out of the 979 participants, 22.7% (n=222/979) reported that they had tested for COVID-19, including 23.0% (n=51/222) who reported positive results, 72.5% (n=161/222) who reported negative results, and 4.5% (n=10/222) who did not know the results at the time of participation. Of the 222 participants who got tested, 44.6% (n=99) had at least one positive case in their immediate family, 45.5% (n=101) had at least one positive case among close friends or relatives, and 51.3% (n=114) had at least one positive case in their local communities.

The extent of receiving COVID-19–related health information online was not associated with their odds of testing (OR 1.03, 95% CI 0.81-1.32, *P*=.79). Awareness of COVID-19 infection in one’s social surroundings was associated with odds of testing, although the patterns slightly differed between close and distant social circles. For participants who had positive cases in their immediate family, their odds of testing were much higher than those who did not have any positive cases in their immediate family (OR 1.48, 95% CI 8.28-26.44, *P*<.001). Similarly, for participants who had any close friends or relatives that tested positive, their odds of testing were much higher compared with those who had no close friends or relatives with positive test results (OR 2.52, 95% CI 1.58-4.03, *P*<.001). Participants who were aware of positive cases in their local communities did not appear to differ in testing from those who were not aware or uncertain if there were any positive cases in local communities.

Results of the logistic regression showed that several demographic characteristics were associated with testing for COVID-19. Specifically, the odds of females getting tested were only 39.75% (95% CI 28.12%-56.20%) of those of males (*P*<.001). The odds of African Americans getting tested were 249.98% (95% CI 156.95%-398.14%) of those of white Americans (*P*<.001) and 275.45% (95% CI 118.73%-639.01%) of those of Asian Americans (*P*=.02). Older participants’ odds of testing were 96.39% (95% CI 94.76%-98.05%) of those who were 1 year younger (*P*<.001). The odds of testing among participants who are married or have domestic partners were 383.92% (95% CI 260.70%-565.36%) of those of singles (*P*<.001).

## Discussion

### Principal Findings

This study examined if internet use, risk awareness, and demographic characteristics were associated with engagement in preventive behaviors and testing for COVID-19 in the United States. Taken as a whole, our data revealed several notable patterns of findings pertaining to preventive behaviors.

First, consistent with findings from other studies on individuals’ engagement in preventive measures against COVID-19 [9,22,23], our data showed that there is a greater tendency for people to engage in some preventative behaviors than others. Although there is evidence showing that, as the pandemic progresses, individuals develop greater awareness of the health risk posed by the virus and engage in protective behaviors with increasing frequency [9], we found that this development is unequal across different types of preventive behaviors. Specifically, people seemed to be more active in adopting the preventive measures of washing hands, covering mouths when sneezing, keeping social distance, and avoiding public transportation than wearing a facemask, staying home, or cleaning frequently touched surfaces. This finding is consistent with several recent studies that assessed the public’s perceptions or knowledge about COVID-19 [9]. In particular, people have different perceptions of the effectiveness or necessity of various preventive measures. For example, although wearing a facemask in public has been a mandatory preventive measure in some countries [23] and many states in the United States, its utility as a preventive measure against the coronavirus is still highly controversial [24]. In addition, as some scholars have noted, the practice of mask wearing is an evolving and cultural phenomenon [10]. Although older people in the United States were found to be less likely to wear a mask during the early stage of the pandemic, which is likely due to their higher knowledge of CDC and NIH recommendations against mask use [10], our survey showed that they seemed to have quickly adapted their behaviors following nationwide revised recommendations for mask use. At the same time, different levels of engagement in preventive behaviors during the COVID-19 pandemic can be attributed to the fact that some preventive measures require greater effort (eg, cleaning frequently touched surfaces) or pose greater difficulties (eg, staying home) than others and are thus deemed less feasible.

Second, we observed positive associations between the extent of receiving COVID-19–related information online and engagement in all types of preventive behaviors. It is possible that a good proportion of the COVID-19–related information people received or sought online involved recommendations on preventive measures [25], leading to more frequent engagement in preventive behaviors. At the same time, this finding suggests that receiving pandemic-related information online, despite vast variation in information content, may enhance people’s concerns about the pandemic and motivate them to actively take preventive measures [26].

Third, risk awareness regarding infection in one’s immediate family, close friends and relatives, and local communities was differentially associated with engagement in preventative behaviors. Compared with participants who did not have positive cases in their immediate family, those who had positive cases in their immediate family reported less frequent engagement in almost all preventive behaviors. Although this finding is somewhat counterintuitive, it also implies that a lack of prevention may lead to higher risk of infection in a family. Immediate family members, as the closest contacts, are at high risk of transmitting the virus within the household. This finding suggests that taking preventive behaviors not only helps one protect themself but also reduces risks of their immediate families becoming infected. Awareness of positive cases among friends and relatives seemed to have limited influence on one’s preventative behaviors. A possible explanation is that many people do not live in close proximity with their friends and relatives, and thus do not perceive high risk of infection from them. One’s local communities appeared to have a positive impact on engagement with preventive behaviors. Compared with participants who reported no positive cases in their communities, those who were aware of positive cases in their communities more frequently washed hands, covered nose and mouth when coughing, kept social distance, and minimized using public transportation. This finding suggests that one’s behavioral responses to a pandemic is influenced by immediate risk factors in one’s surroundings.

Fourth, subgroups tend to differ in their engagement in preventive behaviors. For example, consistent with many past studies, our data showed that being older, female [27-32], or nonwhite [33] is associated with a higher chance of adopting preventive behaviors during a pandemic involving respiratory type diseases. This finding is also consistent with recent research on COVID-19 showing that older people and females were more knowledgeable about COVID-19 [10].

In addition, working people reported less frequent engagement in terms of avoiding public transportation and keeping social distance. This finding suggests that working individuals, especially those who cannot work from home, face greater challenges in implementing certain preventative measures. In particular, for individuals who work in essential businesses (eg, supermarkets, health care, post office, food processing factories), it may not be feasible for them to employ preventive measures such as keeping social distance or avoiding public transportation.

Our findings pertaining to testing behaviors seemed less straightforward. The extent of receiving health information online was not associated with testing. This may be largely due to the fact that during the early stage of the pandemic eligibility criteria for testing were highly stringent, and testing capacity was limited in most parts of the country. Although individuals can request a test for COVID-19, whether or not one will get tested is a decision ultimately made by health departments and health professionals. In other words, unlike many preventive behaviors that can be performed based on one’s own volition, individuals have much less power in decision making regarding testing.

Not surprisingly, individuals who had confirmed COVID-19 cases in their immediate family were more likely to get tested. Individuals who had confirmed COVID-19 cases among their close friends and relatives were also likely to get tested. On the other hand, positive cases in one’s local communities did not seem to have a significant influence on one’s testing. These findings are consistent with CDC’s guidelines for testing, one of which is close contact with patients who are infected. As testing capacity increases and testing criteria become less stringent, it is reasonable to expect that awareness of positive cases in local communities would motivate more individuals to request testing.

Our study also reveals some subgroup differences in testing. For example, we found that working individuals were twice as likely as nonworking individuals to get tested for COVID-19. In addition, individuals who are single were much less likely than those who are married or have domestic partners to get tested. Given that individuals may contract the coronavirus at virtually any setting with others around (eg, grocery stores, parks, social gatherings), this finding suggests that some nonworking individuals or those who live by themselves might have underestimated their risk of COVID-19 infection.

Our findings offer several implications for interventions, communication strategies, and future research. First, in light of recent research showing that many health care workers had poor knowledge of the mode of transmission and the incubation period of COVID-19 [33], both of which can affect health care workers’ recommendations to their patients regarding preventive behaviors and testing (eg, asymptomatic patients often do not get tested), there is an urgent need to provide health care workers with up-to-date information about the disease. Second, as shelter-in-place orders remain in effect in most states in the United States, greater efforts should be put into increasing household internet coverage so that more people will have easy and prompt access to information related to the pandemic [34]. Third, our findings suggest a need for more public health education programs and interventions targeting certain subgroups that have consistently shown to be less likely to adopt preventive measures during a pandemic. Identifying more effective strategies that can be used to induce self-protective behaviors in groups such as young males, for example, can help slow down the spread of the virus. At the same time, as other scholars have noted, although demographic characteristics are generally immutable, future research needs to obtain a deeper understanding of the root causes of differential behavioral responses, which can help inform the development of dissemination strategies directed at different subgroups [28].

### Limitations

This study has several limitations. First, given the cross-sectional nature of our data, we are not in a position to empirically assess and demonstrate causal relationships among the variables. For instance, confirmed cases in one’s social surroundings may prompt people to engage in more active preventive behaviors, and one’s engagement in preventive behaviors during a pandemic like COVID-19 can certainly influence the health of families, friends, and people in local communities.

Second, given the urgency of the research needs and limited access to nationally representative samples, we elected to recruit our participants from an online crowdsourcing platform. Although the composition of ethnicity, sex, and marital status in our data largely mirrored the demographics profile in the US population [35-37], our sample included more young, educated, and working participants. Ideally, a randomly selected sample of the public should be surveyed and comparisons made with the known distribution of key variables in the population.

Third, although social desirability bias has been found to be lower in anonymous online surveys than in telephone or in-person surveys [38], we cannot rule out the possibility of some response bias in the self-reported data. In particular, potential group variations in response bias may have influenced some findings. For example, past research suggests that females tend to show more social desirability than males in survey responses [39]. Although the observed pattern of gender differences in this study is largely consistent with past research, we still cannot rule out the possibility that female participants reported more frequent engagement in preventive behaviors than male participants due, in part, to social desirability. Despite this limitation, a recent study [4] has validated the use of rapid surveys to examine COVID-19–related perceptions and behaviors.

### Conclusions

During the ongoing and rapidly evolving COVID-19 pandemic, we have seen a bourgeoning amount of research pertaining to the pathophysiology, diagnosis, and treatment of the novel disease, which is urgently and rightfully needed. Research examining the public’s behavioral (and cognitive, psychological) responses to the pandemic, however, also deserves attention, as it can help inform formulation and implementation of public health policies and control measures. This study offers useful insights into factors that are associated with engagement in preventive behaviors and testing of COVID-19. Our findings revealed that the extent of receiving COVID-19–related information online, risk awareness, and demographic characteristics including sex, ethnicity, age, marital status, and employment status are key factors associated with individuals’ engagement in various preventive behaviors and testing for COVID-19.

## Appendix

Multimedia Appendix 1 Binomial and ordinal logistic regression results on testing and engagement in preventive behaviors.

Multimedia Appendix 2 Simplified questionnaire.

## Abbreviations

CDC: Centers for Disease Control and Prevention
COVID-19: coronavirus disease
MTurk: Amazon Mechanical Turk
NIH: National Institute of Health
OR: odds ratio
