# Supplementary material for: Internet Use, Risk Awareness, and Demographic Characteristics Associated With Engagement in Preventive Behaviors and Testing: Cross-Sectional Survey on COVID-19 in the United States
Source: J Med Internet Res. 2020 Jun 16;22(6):e19782. doi: 10.2196/19782 (PMC7299540; doi:10.2196/19782)
Supplement: Multimedia Appendix 1 [file jmir_v22i6e19782_app1.docx]

Table 1. Logistic regression results on getting tested for COVID-19

| Predictors |  | Model 1 | | |  | Model 2 | | |  | | Model 3 | | | | | | |
| --- | --- | --- | --- | --- | --- | --- | --- | --- | --- | --- | --- | --- | --- | --- | --- | --- | --- |
|  | *B* | *Exp (B)* | *Wald-stat* | *P- value* | *B* | *Exp (B)* | *Wald-stat* | *P- value* | *B* | *Exp (B)* | | *Wald-stat* | | *P- value* | |  |  |
| Sex | -0.92 | 0.40 | 27.27 | <.001 | -0.88 | 0.42 | 23.96 | <.001 | -0.86 | 0.42 | | | 17.57 | | <.001 | |  |
| Age | -0.04 | 0.96 | 17.8 | <.001 | -0.04 | 0.96 | 17.59 | <.001 | -0.04 | 0.96 | | | 13.99 | | <.001 | |  |
| Ethnicity: Black | 0.92 | 2.50 | 14.88 | <.001 | 0.77 | 2.16 | 9.88 | <.01 | 0.66 | 1.93 | | | 5.15 | | .02 | |  |
| Ethnicity: Hispanic | 0.63 | 1.87 | 3.15 | .08 | 0.60 | 1.82 | 2.77 | .10 | 0.22 | 1.24 | | | 0.25 | | .62 | |  |
| Ethnicity: Asian | -0.63 | 0.53 | 3.66 | .06 | -0.64 | 0.53 | 3.64 | .06 | -0.39 | 0.68 | | | 1.17 | | .28 | |  |
| Ethnicity: Other | 0.13 | 1.13 | 0.08 | .77 | 0.14 | 1.15 | 0.11 | .75 | -0.08 | 0.92 | | | 0.03 | | .87 | |  |
| Education | 0.08 | 1.09 | 3.70 | .05 | 0.08 | 1.08 | 3.10 | .08 | 0.06 | 1.06 | | | 1.28 | | .26 | |  |
| Log (Income) | -0.20 | 0.82 | 2.47 | .12 | -0.13 | 0.88 | 0.98 | .32 | -0.07 | 0.94 | | | 0.18 | | .67 | |  |
| Employment Status | 0.76 | 2.14 | 6.62 | .01 | 0.76 | 2.14 | 6.37 | .01 | 0.62 | 1.85 | | | 3.48 | | .06 | |  |
| Marital Status | 1.35 | 3.84 | 46.41 | <.001 | 1.30 | 3.68 | 42.24 | <.001 | 1.24 | 3.45 | | | 28.74 | | <.001 | |  |
| Time Spent Online |  |  |  |  | 0.08 | 1.08 | 21.11 | <.001 | 0.05 | 1.05 | | | 6.33 | | .01 | |  |
| Received  Information Online |  |  |  |  | 0.09 | 1.09 | 0.68 | .41 | 0.03 | 1.03 | | | 0.07 | | .79 | |  |
| Community Positive |  |  |  |  |  |  |  |  | -0.25 | 0.78 | | | 1.27 | | .26 | |  |
| Community Unknown |  |  |  |  |  |  |  |  | -1.18 | 0.31 | | | 11.88 | | <.001 | |  |
| Family Positive |  |  |  |  |  |  |  |  | 2.69 | 14.80 | | | 82.83 | | <.001 | |  |
| Family Unknown |  |  |  |  |  |  |  |  | 1.17 | 1.26 | | | 3.37 | | .07 | |  |
| Friends Positive |  |  |  |  |  |  |  |  | 0.93 | 2.52 | | | 14.92 | | <.001 | |  |
| Friends Unknown |  |  |  |  |  |  |  |  | 0.23 | 1.26 | | | 1.26 | | .68 | |  |
| Model Chi-square | 162.85 |  |  |  | 185.72 |  |  |  | 360.79 |  | | |  | |  | |  |
| Block Chi-square |  |  |  |  | 22.87 |  |  |  | 175.08 |  | | |  | |  | |  |
| % Correct Predictions | 79.57 |  |  |  | 80.08 |  |  |  | 85.90 |  | | |  | |  | |  |
| Nagelkerke R Square | 0.23 |  |  |  | 0.26 |  |  |  | 0.47 |  | | |  | |  | |  |

Table 2. Ordinal logistic regression results on wearing masks

| Predictors |  | Model 1 | | |  | Model 2 | | |  | | Model 3 | | | | | | |
| --- | --- | --- | --- | --- | --- | --- | --- | --- | --- | --- | --- | --- | --- | --- | --- | --- | --- |
|  | *B* | *Exp (B)* | *Wald-stat* | *P- value* | *B* | *Exp (B)* | *Wald-stat* | *P- value* | *B* | *Exp (B)* | | *Wald-stat* | | *P- value* | |  |  |
| Sex | 0.00 | 1.00 | 0.23 | .99 | -0.02 | 0.98 | 0.02 | .88 | 0.02 | 1.02 | | | 0.02 | | .89 | |  |
| Age | -0.01 | 0.99 | 2.57 | .11 | -0.01 | 0.99 | 3.48 | .06 | -0.01 | 0.99 | | | 2.94 | | .09 | |  |
| Ethnicity: Black | 0.59 | 1.81 | 10.31 | .001 | 0.46 | 1.59 | 6.25 | .012 | 0.46 | 1.59 | | | 6.15 | | .01 | |  |
| Ethnicity: Hispanic | 0.26 | 1.30 | 0.95 | .32 | 0.30 | 1.34 | 1.22 | .27 | 0.24 | 1.27 | | | 0.77 | | .38 | |  |
| Ethnicity: Asian | 0.90 | 2.47 | 19.30 | <.001 | 0.93 | 2.53 | 19.73 | <.001 | 0.99 | 2.69 | | | 21.97 | | <.001 | |  |
| Ethnicity: Other | 0.58 | 1.79 | 4.39 | .04 | 0.58 | 1.78 | 4.34 | .04 | 0.45 | 1.58 | | | 2.59 | | .11 | |  |
| Education | 0.00 | 1.00 | 0.00 | .99 | -0.01 | 0.99 | 0.11 | .74 | -.02 | 0.99 | | | 0.24 | | .62 | |  |
| Log (Income) | -0.06 | 0.95 | 0.46 | .50 | -0.07 | 0.94 | 0.62 | .43 | -0.03 | 0.97 | | | 0.15 | | .70 | |  |
| Employment Status | 0.18 | 1.19 | 1.22 | .27 | 0.10 | 1.10 | 0.35 | .55 | 0.11 | 1.11 | | | 0.41 | | .52 | |  |
| Marital Status | 0.36 | 1.43 | 7.91 | .005 | 0.36 | 1.43 | 7.86 | .005 | 0.32 | 1.37 | | | 6.02 | | .01 | |  |
| Time Spent Online |  |  |  |  | 0.01 | 1.01 | 0.96 | .33 | 0.01 | 1.01 | | | 0.37 | | .55 | |  |
| Received  Information Online |  |  |  |  | 0.44 | 1.56 | 37.88 | <.001 | 0.44 | 1.34 | | | 35.22 | | <.001 | |  |
| Community Positive |  |  |  |  |  |  |  |  | -0.07 | 0.72 | | | 0.24 | | .62 | |  |
| Community Unknown |  |  |  |  |  |  |  |  | 0.10 | 0.78 | | | 0.31 | | .58 | |  |
| Friends Positive |  |  |  |  |  |  |  |  | 0.29 | 0.98 | | | 3.32 | | .07 | |  |
| Friends Unknown |  |  |  |  |  |  |  |  | 0.27 | 0.670 | | | 0.71 | | .40 | |  |
| Family Positive |  |  |  |  |  |  |  |  | 0.25 | 0.88 | | | 1.65 | | .20 | |  |
| Family Unknown |  |  |  |  |  |  |  |  | 0.84 | 0.99 | | | 3.70 | | .05 | |  |
| Model Chi-square | 41.92 |  |  |  | 82.90 |  |  |  | 98.03 |  | | |  | |  | |  |
| Degree of freedom | 10 |  |  |  | 12 |  |  |  | 18 |  | | |  | |  | |  |
| Model significance | <.001 |  |  |  | <.001 |  |  |  | <.001 |  | | |  | |  | |  |

Table 3. Ordinal logistic regression results on washing hands

| Predictors |  | Model 1 | | |  | Model 2 | | |  | | Model 3 | | | | | | |
| --- | --- | --- | --- | --- | --- | --- | --- | --- | --- | --- | --- | --- | --- | --- | --- | --- | --- |
|  | *B* | *Exp (B)* | *Wald-stat* | *P- value* | *B* | *Exp (B)* | *Wald-stat* | *P- value* | *B* | *Exp (B)* | | *Wald-stat* | | *P- value* | |  |  |
| Sex | 0.87 | 2.39 | 44.43 | <.001 | 0.82 | 2.28 | 38.42 | <.001 | 0.78 | 2.18 | | | 33.13 | | <.001 | |  |
| Age | 0.01 | 1.01 | 4.10 | .04 | 0.01 | 1.01 | 3.26 | .07 | -0.01 | 1.01 | | | 2.20 | | .14 | |  |
| Ethnicity: Black | 0.18 | 1.19 | 0.79 | .38 | 0.18 | 1.20 | 0.79 | .37 | 0.31 | 1.36 | | | 2.15 | | .14 | |  |
| Ethnicity: Hispanic | 0.01 | 1.01 | 0.00 | .98 | 0.14 | 1.15 | 0.20 | .65 | 0.20 | 1.23 | | | 0.45 | | .51 | |  |
| Ethnicity: Asian | 0.36 | 1.43 | 2.64 | .10 | 0.40 | 1.49 | 3.15 | .07 | 0.31 | 1.35 | | | 1.83 | | .17 | |  |
| Ethnicity: Other | 0.13 | 1.14 | 0.19 | .66 | 0.10 | 1.10 | 0.10 | .75 | 0.05 | 1.05 | | | 0.02 | | .88 | |  |
| Education | -0.07 | 0.93 | 4.98 | .03 | -0.08 | 0.93 | 5.36 | .02 | -.07 | 0.93 | | | 4.74 | | .03 | |  |
| Log (Income) | 0.10 | 1.10 | 1.09 | .28 | 0.05 | 1.05 | 0.26 | .61 | -0.02 | 1.02 | | | 0.04 | | .85 | |  |
| Employment Status | -0.32 | 0.73 | 3.08 | .08 | -0.44 | 0.64 | 5.65 | .02 | -0.35 | 0.70 | | | 3.54 | | .06 | |  |
| Marital Status | -0.20 | 0.82 | 2.07 | .15 | -0.18 | 0.83 | 1.70 | .19 | -0.10 | 0.91 | | | 0.47 | | .49 | |  |
| Time Spent Online |  |  |  |  | -0.04 | 0.96 | 10.52 | .001 | -0.03 | 0.97 | | | 4.61 | | .03 | |  |
| Received  Information Online |  |  |  |  | 0.45 | 1.57 | 33.87 | <.001 | 0.46 | 1.58 | | | 33.27 | | <.001 | |  |
| Community Positive |  |  |  |  |  |  |  |  | 0.43 | 1.54 | | | 8.48 | | .004 | |  |
| Community Unknown |  |  |  |  |  |  |  |  | 0.64 | 1.90 | | | 10.91 | | <.001 | |  |
| Friends Positive |  |  |  |  |  |  |  |  | 0.21 | 1.23 | | | 1.35 | | .26 | |  |
| Friends Unknown |  |  |  |  |  |  |  |  | -0.33 | 0.72 | | | 0.85 | | .36 | |  |
| Family Positive |  |  |  |  |  |  |  |  | -1.05 | 0.35 | | | 25.78 | | <.001 | |  |
| Family Unknown |  |  |  |  |  |  |  |  | -0.73 | 0.48 | | | 2.64 | | .10 | |  |
| Model Chi-square | 72.81 |  |  |  | 113.90 |  |  |  | 156.02 |  | | |  | |  | |  |
| Degree of freedom | 10 |  |  |  | 12 |  |  |  | 18 |  | | |  | |  | |  |
| Model significance | <.001 |  |  |  | <.001 |  |  |  | <.001 |  | | |  | |  | |  |

Table 4. Ordinal logistic regression results on covering nose and mouth when coughing or sneezing

| Predictors |  | Model 1 | | |  | Model 2 | | |  | | Model 3 | | | | | | |
| --- | --- | --- | --- | --- | --- | --- | --- | --- | --- | --- | --- | --- | --- | --- | --- | --- | --- |
|  | *B* | *Exp (B)* | *Wald-stat* | *P- value* | *B* | *Exp (B)* | *Wald-stat* | *P- value* | *B* | *Exp (B)* | | *Wald-stat* | | *P- value* | |  |  |
| Sex | 0.75 | 2.12 | 31.76 | <.001 | 0.70 | 2.02 | 26.78 | <.001 | 0.66 | 1.93 | | | 22.94 | | <.001 | |  |
| Age | 0.01 | 1.01 | 5.62 | .018 | 0.01 | 1.01 | 3.90 | .048 | 0.01 | 1.01 | | | 3.02 | | .08 | |  |
| Ethnicity: Black | 0.37 | 1.45 | 0.07 | <.001 | 0.37 | 1.45 | 3.08 | .08 | 0.46 | 1.58 | | | 4.53 | | .03 | |  |
| Ethnicity: Hispanic | 0.14 | 1.15 | 0.65 | .08 | 0.25 | 1.29 | 0.64 | .42 | 0.28 | 1.33 | | | 0.80 | | .37 | |  |
| Ethnicity: Asian | 0.58 | 1.78 | 0.013 | .06 | 0.66 | 1.93 | 7.67 | .006 | 0.57 | 1.77 | | | 5.82 | | .02 | |  |
| Ethnicity: Other | 0.34 | 1.40 | 0.29 | .77 | 0.30 | 1.36 | 0.88 | .35 | 0.33 | 1.39 | | | 0.96 | | .32 | |  |
| Education | -0.14 | 0.87 | 17.21 | <.001 | -0.16 | 0.86 | 19.97 | <.001 | -0.15 | 0.86 | | | 18.87 | | <.001 | |  |
| Log (Income) | 0.22 | 1.25 | 5.63 | .018 | 0.16 | 1.18 | 2.94 | .09 | 0.14 | 1.15 | | | 2.11 | | .15 | |  |
| Employment Status | -0.28 | 0.76 | 0.14 | .01 | -0.45 | 0.64 | 5.24 | .02 | -0.36 | 0.70 | | | 3.29 | | .07 | |  |
| Marital Status | -0.17 | 0.84 | 0.23 | <.001 | -0.13 | 0.88 | 0.82 | .37 | -0.07 | 0.92 | | | 0.25 | | .61 | |  |
| Time Spent Online |  |  |  |  | -0.06 | 0.95 | 16.40 | <.001 | -0.04 | 0.96 | | | 9.55 | | .002 | |  |
| Received  Information Online |  |  |  |  | 0.60 | 1.82 | 53.91 | <.001 | 0.58 | 1.78 | | | 48.95 | | <.001 | |  |
| Community Positive |  |  |  |  |  |  |  |  | 0.52 | 1.68 | | | 11.73 | | .001 | |  |
| Community Unknown |  |  |  |  |  |  |  |  | 0.50 | 1.64 | | | 6.41 | | .01 | |  |
| Friends Positive |  |  |  |  |  |  |  |  | 0.03 | 1.03 | | | 0.02 | | .88 | |  |
| Friends Unknown |  |  |  |  |  |  |  |  | -0.06 | 0.94 | | | 0.03 | | .86 | |  |
| Family Positive |  |  |  |  |  |  |  |  | -0.64 | 0.53 | | | 9.07 | | .003 | |  |
| Family Unknown |  |  |  |  |  |  |  |  | -0.82 | 0.44 | | | 3.02 | | .08 | |  |
| Model Chi-square | 75.91 |  |  |  | 141.97 |  |  |  | 165.67 |  | | |  | |  | |  |
| Degree of freedom | 10 |  |  |  | 12 |  |  |  | 18 |  | | |  | |  | |  |
| Model significance | <.001 |  |  |  | <.001 |  |  |  | <.001 |  | | |  | |  | |  |

Table 5. Ordinal logistic regression results on keeping social distance

| Predictors |  | Model 1 | | |  | Model 2 | | |  | | Model 3 | | | | | | |
| --- | --- | --- | --- | --- | --- | --- | --- | --- | --- | --- | --- | --- | --- | --- | --- | --- | --- |
|  | *B* | *Exp (B)* | *Wald-stat* | *P- value* | *B* | *Exp (B)* | *Wald-stat* | *P- value* | *B* | *Exp (B)* | | *Wald-stat* | | *P- value* | |  |  |
| Sex | 0.49 | 1.64 | 14.90 | <.001 | 0.46 | 1.58 | 12.61 | <.001 | 0.42 | 1.53 | | | 10.34 | | .001 | |  |
| Age | 0.02 | 1.02 | 10.50 | .001 | 0.02 | 1.02 | 9.53 | .002 | 0.02 | 1.02 | | | 8.27 | | .004 | |  |
| Ethnicity: Black | 0.36 | 1.44 | 3.17 | .08 | 0.34 | 1.40 | 2.71 | .10 | 0.49 | 1.63 | | | 5.24 | | .02 | |  |
| Ethnicity: Hispanic | -0.24 | 0.78 | 0.71 | .40 | -0.20 | 0.82 | 0.47 | .50 | -0.11 | 0.90 | | | 0.13 | | .72 | |  |
| Ethnicity: Asian | 0.48 | 1.61 | 4.64 | .03 | 0.51 | 1.66 | 5.17 | .02 | 0.46 | 1.58 | | | 4.18 | | .04 | |  |
| Ethnicity: Other | -0.16 | 0.86 | 0.27 | .60 | -0.19 | 0.83 | 0.41 | .52 | -0.31 | 0.73 | | | 1.03 | | .31 | |  |
| Education | 0.08 | 1.09 | 3.70 | .05 | -0.11 | 0.89 | 12.23 | <.001 | -0.11 | 0.89 | | | 11.50 | | .001 | |  |
| Log (Income) | -0.20 | 0.82 | 2.47 | .12 | 0.11 | 1.12 | 1.52 | .22 | 0.11 | 1.12 | | | 1.36 | | .24 | |  |
| Employment Status | -0.11 | 0.90 | 11.22 | .001 | -0.79 | 0.45 | 18.35 | <.001 | -0.07 | 0.49 | | | 13.98 | | <.001 | |  |
| Marital Status | 0.15 | 1.16 | 2.65 | .10 | -0.09 | 0.92 | 0.38 | .54 | -0.03 | 0.98 | | | 0.3 | | .86 | |  |
| Time Spent Online |  |  |  |  | -0.02 | 0.98 | 3.33 | .07 | -0.01 | 0.99 | | | 0.87 | | .35 | |  |
| Received  Information Online |  |  |  |  | 0.35 | 1.42 | 21.08 | <.001 | 0.35 | 1.41 | | | 19.95 | | <.001 | |  |
| Community Positive |  |  |  |  |  |  |  |  | 0.41 | 1.51 | | | 7.90 | | .005 | |  |
| Community Unknown |  |  |  |  |  |  |  |  | 0.74 | 2.10 | | | 14.65 | | <.001 | |  |
| Friends Positive |  |  |  |  |  |  |  |  | 0.29 | 1.33 | | | 2.60 | | .11 | |  |
| Friends Unknown |  |  |  |  |  |  |  |  | -0.18 | 0.83 | | | 0.24 | | .63 | |  |
| Family Positive |  |  |  |  |  |  |  |  | -0.92 | 0.40 | | | 18.60 | | <.001 | |  |
| Family Unknown |  |  |  |  |  |  |  |  | -0.35 | 0.70 | | | 0.52 | | .47 | |  |
| Model Chi-square | 72.33 |  |  |  | 95.41 |  |  |  | 130.68 |  | | |  | |  | |  |
| Degree of freedom | 10 |  |  |  | 12 |  |  |  | 18 |  | | |  | |  | |  |
| Model significance |  |  |  |  | <.001 |  |  |  | <.001 |  | | |  | |  | |  |

Table 6. Ordinal logistic regression results on staying home

| Predictors |  | Model 1 | | |  | Model 2 | | |  | | Model 3 | | | | | | |
| --- | --- | --- | --- | --- | --- | --- | --- | --- | --- | --- | --- | --- | --- | --- | --- | --- | --- |
|  | *B* | *Exp (B)* | *Wald-stat* | *P- value* | *B* | *Exp (B)* | *Wald-stat* | *P- value* | *B* | *Exp (B)* | | *Wald-stat* | | *P- value* | |  |  |
| Sex | 0.29 | 1.34 | 5.54 | .019 | 0.28 | 1.32 | 4.94 | .026 | 0.26 | 1.30 | | | 4.43 | | .04 | |  |
| Age | 0.01 | 1.01 | 5.52 | .019 | 0.01 | 1.01 | 4.87 | .027 | 0.1 | 1.01 | | | 4.96 | | .03 | |  |
| Ethnicity: Black | 0.63 | 1.88 | 10.17 | .001 | 0.55 | 1.73 | 7.54 | .006 | 0.59 | 1.80 | | | 8.42 | | .004 | |  |
| Ethnicity: Hispanic | 0.20 | 1.22 | 0.46 | .50 | 0.25 | 1.28 | 0.70 | .40 | 0.27 | 1.31 | | | 0.81 | | .37 | |  |
| Ethnicity: Asian | 0.80 | 2.23 | 14.26 | <.001 | 0.84 | 2.31 | 15.24 | <.001 | 0.83 | 2.29 | | | 14.76 | | <.001 | |  |
| Ethnicity: Other | 0.02 | 1.02 | 0.01 | .94 | 0.01 | 1.01 | 0.00 | .96 | -0.05 | 0.95 | | | 0.04 | | .85 | |  |
| Education | -0.06 | 0.95 | 3.17 | .08 | -0.06 | 0.94 | 3.79 | .05 | -0.06 | 0.94 | | | 3.64 | | .06 | |  |
| Log (Income) | -0.03 | 0.97 | 0.16 | .69 | -0.05 | 0.95 | 0.34 | .56 | -0.05 | 0.95 | | | 0.29 | | .59 | |  |
| Employment Status | -0.26 | 0.77 | 2.66 | .10 | -0.34 | 0.71 | 4.30 | .04 | -0.28 | 0.75 | | | 2.89 | | .09 | |  |
| Marital Status | -0.12 | 0.89 | 0.79 | .37 | -0.11 | 0.89 | 0.74 | .40 | -0.11 | 0.90 | | | 0.63 | | .43 | |  |
| Time Spent Online |  |  |  |  | 0.01 | 1.01 | 0.22 | .64 | 0.01 | 1.01 | | | 0.52 | | .52 | |  |
| Received  Information Online |  |  |  |  | 0.34 | 1.41 | 21.94 | <.001 | 0.33 | 1.40 | | | 19.80 | | <.001 | |  |
| Community Positive |  |  |  |  |  |  |  |  | 0.19 | 1.21 | | | 1.87 | | .17 | |  |
| Community Unknown |  |  |  |  |  |  |  |  | 0.28 | 1.32 | | | 2.37 | | .12 | |  |
| Friends Positive |  |  |  |  |  |  |  |  | 0.33 | 1.39 | | | 3.87 | | .049 | |  |
| Friends Unknown |  |  |  |  |  |  |  |  | 0.03 | 1.03 | | | 0.01 | | .93 | |  |
| Family Positive |  |  |  |  |  |  |  |  | -0.39 | 0.67 | | | 3.47 | | .06 | |  |
| Family Unknown |  |  |  |  |  |  |  |  | -0.11 | 0.90 | | | 0.05 | | .82 | |  |
| Model Chi-square | 38.57 |  |  |  | 61.62 |  |  |  | 70.70 |  | | |  | |  | |  |
| Degree of freedom | 10 |  |  |  | 12 |  |  |  | 18 |  | | |  | |  | |  |
| Model significance | <.001 |  |  |  | <.001 |  |  |  | <.001 |  | | |  | |  | |  |

Table 7. Ordinal logistic regression results on avoiding public transportation

| Predictors |  | Model 1 | | |  | Model 2 | | |  | | Model 3 | | | | | | |
| --- | --- | --- | --- | --- | --- | --- | --- | --- | --- | --- | --- | --- | --- | --- | --- | --- | --- |
|  | *B* | *Exp (B)* | *Wald-stat* | *P- value* | *B* | *Exp (B)* | *Wald-stat* | *P- value* | *B* | *Exp (B)* | | *Wald-stat* | | *P- value* | |  |  |
| Sex | 0.83 | 2.30 | 31.67 | <.001 | 0.79 | 2.20 | 27.37 | <.001 | 0.74 | 2.10 | | | 23.25 | | <.001 | |  |
| Age | 0.02 | 1.02 | 12.62 | <.001 | 0.02 | 1.02 | 11.61 | .001 | 0.02 | 1.02 | | | 8.21 | | .004 | |  |
| Ethnicity: Black | -0.17 | 0.84 | 0.68 | .41 | -0.16 | 0.85 | 0.61 | .44 | -0.04 | 0.96 | | | 0.03 | | .86 | |  |
| Ethnicity: Hispanic | -0.53 | 0.59 | 3.07 | .08 | -0.46 | 0.63 | 2.31 | .13 | -0.47 | 0.62 | | | 2.36 | | .13 | |  |
| Ethnicity: Asian | 0.41 | 1.51 | 2.64 | .10 | 0.45 | 1.57 | 3.08 | .08 | 0.32 | 1.38 | | | 1.54 | | .22 | |  |
| Ethnicity: Other | -0.49 | 0.61 | 2.38 | .12 | -0.58 | 0.56 | 3.20 | .07 | -0.63 | 0.53 | | | 3.53 | | .06 | |  |
| Education | -0.12 | 0.88 | 11.25 | .001 | -0.13 | 0.88 | 12.10 | .001 | -0.12 | 0.89 | | | 9.14 | | .003 | |  |
| Log (Income) | 0.26 | 1.29 | 6.32 | .012 | 0.20 | 1.22 | 3.52 | .06 | 0.17 | 1.18 | | | 2.46 | | .12 | |  |
| Employment Status | -0.59 | 0.56 | 6.80 | .009 | -0.72 | 0.49 | 9.69 | .002 | -0.59 | 0.55 | | | 6.29 | | .01 | |  |
| Marital Status | -0.46 | 0.63 | 8.77 | .003 | -0.44 | 0.64 | 7.76 | .005 | -0.35 | 0.70 | | | 4.57 | | .03 | |  |
| Time Spent Online |  |  |  |  | -0.06 | 0.94 | 16.08 | <.001 | -0.04 | 0.96 | | | 6.37 | | .01 | |  |
| Received  Information Online |  |  |  |  | 0.44 | 1.56 | 25.70 | <.001 | 0.45 | 1.57 | | | 25.13 | | <.001 | |  |
| Community Positive |  |  |  |  |  |  |  |  | 0.54 | 1.71 | | | 10.50 | | .001 | |  |
| Community Unknown |  |  |  |  |  |  |  |  | 0.97 | 2.65 | | | 17.42 | | <.001 | |  |
| Friends Positive |  |  |  |  |  |  |  |  | -0.15 | 0.86 | | | 0.59 | | .44 | |  |
| Friends Unknown |  |  |  |  |  |  |  |  | 0.49 | 1.63 | | | 1.04 | | .31 | |  |
| Family Positive |  |  |  |  |  |  |  |  | -0.86 | 0.42 | | | 15.46 | | <.001 | |  |
| Family Unknown |  |  |  |  |  |  |  |  | -1.91 | 0.15 | | | 14.63 | | <.001 | |  |
| Model Chi-square | 104.07 |  |  |  | 141.87 |  |  |  | 191.70 |  | | |  | |  | |  |
| Degree of freedom | 10 |  |  |  | 12 |  |  |  | 18 |  | | |  | |  | |  |
| Model significance | <.001 |  |  |  | <.001 |  |  |  | <.001 |  | | |  | |  | |  |

Table 8. Ordinal logistic regression results on cleaning and disinfecting surfaces

| Predictors |  | Model 1 | | |  | Model 2 | | |  | | Model 3 | | | | | | |
| --- | --- | --- | --- | --- | --- | --- | --- | --- | --- | --- | --- | --- | --- | --- | --- | --- | --- |
|  | *B* | *Exp (B)* | *Wald-stat* | *P- value* | *B* | *Exp (B)* | *Wald-stat* | *P- value* | *B* | *Exp (B)* | | *Wald-stat* | | *P- value* | |  |  |
| Sex | 0.46 | 1.58 | 14.21 | <.001 | 0.42 | 1.53 | 11.88 | .001 | 0.45 | 1.58 | | | 13.43 | | <.001 | |  |
| Age | -0.01 | 0.99 | 1.39 | .24 | -0.01 | 0.99 | 1.88 | .17 | -0.01 | 0.99 | | | 1.59 | | .21 | |  |
| Ethnicity: Black | 0.69 | 2.00 | 12.48 | <.001 | 0.60 | 1.82 | 9.06 | .003 | 0.56 | 1.76 | | | 7.87 | | .005 | |  |
| Ethnicity: Hispanic | 0.44 | 1.55 | 2.48 | .12 | 0.48 | 1.61 | 2.89 | .09 | 0.45 | 1.56 | | | 2.45 | | .12 | |  |
| Ethnicity: Asian | -0.04 | 0.96 | 0.03 | .86 | -0.01 | 0.99 | 0.00 | .96 | 0.03 | 1.03 | | | 0.03 | | .87 | |  |
| Ethnicity: Other | -0.08 | 0.92 | 0.08 | .77 | -0.08 | 0.92 | 0.08 | .78 | -0.18 | 0.84 | | | 0.37 | | .54 | |  |
| Education | -0.07 | 0.93 | 4.81 | .03 | -0.08 | 0.93 | 5.94 | .02 | -0.08 | 0.92 | | | 6.51 | | .01 | |  |
| Log (Income) | 0.02 | 1.02 | 0.07 | .79 | 0.01 | 1.01 | 0.02 | .88 | 0.03 | 1.03 | | | 0.11 | | .74 | |  |
| Employment Status | 0.13 | 1.14 | 0.69 | .41 | 0.02 | 1.02 | 0.02 | .89 | 0.02 | 1.02 | | | 0.02 | | .90 | |  |
| Marital Status | 0.61 | 1.84 | 21.26 | <.001 | 0.62 | 1.86 | 22.04 | <.001 | 0.59 | 1.80 | | | 19.37 | | <.001 | |  |
| Time Spent Online |  |  |  |  | 0.00 | 1.00 | 0.14 | .71 | 0.00 | 1.00 | | | 0.00 | | .96 | |  |
| Received  Information Online |  |  |  |  | 0.44 | 1.55 | 36.12 | <.001 | 0.44 | 1.55 | | | 34.77 | | <.001 | |  |
| Community Positive |  |  |  |  |  |  |  |  | -0.12 | 0.89 | | | 0.71 | | .40 | |  |
| Community Unknown |  |  |  |  |  |  |  |  | 0.05 | 1.05 | | | 0.08 | | .77 | |  |
| Friends Positive |  |  |  |  |  |  |  |  | 0.42 | 1.52 | | | 6.48 | | .01 | |  |
| Friends Unknown |  |  |  |  |  |  |  |  | -0.16 | 0.85 | | | 0.23 | | .63 | |  |
| Family Positive |  |  |  |  |  |  |  |  | 0.11 | 1.12 | | | 0.32 | | .57 | |  |
| Family Unknown |  |  |  |  |  |  |  |  | 0.55 | 1.74 | | | 1.44 | | .23 | |  |
| Model Chi-square | 54.04 |  |  |  | 91.95 |  |  |  | 103.12 |  | | |  | |  | |  |
| Degree of freedom | 10 |  |  |  | 12 |  |  |  | 18 |  | | |  | |  | |  |
| Model significance | <.001 |  |  |  | <.001 |  |  |  | <.001 |  | | |  | |  | |  |
