# Supplementary material for: Internet Use, Risk Awareness, and Demographic Characteristics Associated With Engagement in Preventive Behaviors and Testing: Cross-Sectional Survey on COVID-19 in the United States
Source: J Med Internet Res. 2020 Jun 16;22(6):e19782. doi: 10.2196/19782 (PMC7299540; doi:10.2196/19782)
Supplement: Multimedia Appendix 2 [file jmir_v22i6e19782_app2.docx]

Survey questionnaire

Below is a simplified version of the survey including all variables examined in this study. The actual survey includes more measures which were not the focus of this study and were thus not reported here.

Q1 Have you already been tested for COVID-19?

- Yes
- No

Display This Question:

If Have you already been tested for COVID-19? = Yes

Q2 If yes, what is your test results?

- Positive
- Negative
- Don't know

Q3 Has anybody in your **immediate family (e.g., children, spouse, siblings, pare**nts) been tested positive for COVID-19?

- Yes
- No
- Don't know

Q4 Has any of your **close friends or relatives (not including members of your immediate family**) been tested positive for COVID-19?

- Yes
- No
- Don't know

Q5 Has anybody in your **local community (e.g., school, workplace, church)** been tested positive for COVID-19?

- Yes
- No
- Don't know

|  |
| --- |

Q6 Over the past month, how much time (in hours) have you spent **online** on a daily basis?

|  | 0 | 1 | 2 | 3 | 4 | 5 | 6 | 7 | 8 | 9 | 10 | 11 | 12 | 13 | 14 | 15 | 16 | 17 | 18 | 19 | 20 |
| --- | --- | --- | --- | --- | --- | --- | --- | --- | --- | --- | --- | --- | --- | --- | --- | --- | --- | --- | --- | --- | --- |

| Hour () | 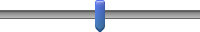 |
| --- | --- |

Q7 Over the past month, to what extent have you received the following types of **information** online?

|  | Didn't Receive at All (1) | Received Rarely (2) | Received Occasionally (3) | Received Regularly (4) | Received a Great Deal (5) |
| --- | --- | --- | --- | --- | --- |
| (1) Information regarding the scientific facts (e.g., symptoms, causes of the disease) related to the pandemic |  |  |  |  |  |
| (2) Information regarding how to prevent contracting the virus |  |  |  |  |  |
| (3) Information regarding the spreading of the virus |  |  |  |  |  |
| (4) Information regarding the sources and resources to give and receive social support during the pandemic |  |  |  |  |  |

Q8 Over the past month, how often have you engaged in the following practices to minimize the risk of contracting the coronavirus (COVID-19)?

|  | Never (1) | Sometimes (2) | About half the time (3) | Most of the time (4) | Always (5) |
| --- | --- | --- | --- | --- | --- |
| (1) Wear a facemask in public even if I am not sick |  |  |  |  |  |
| (2) Wash hands regularly for 20 seconds, with soap and water or alcohol-based hand rub |  |  |  |  |  |
| (3) Cover nose and mouth with a disposable tissue or flexed elbow when cough or sneeze |  |  |  |  |  |
| (4) Keep safe social distance with others |  |  |  |  |  |
| (5) Stay home |  |  |  |  |  |
| (6) Avoid using public transportation |  |  |  |  |  |
| (7) Clean and disinfect frequently touched surfaces such as doorknobs, phones, and keyboards daily |  |  |  |  |  |

|  |
| --- |

Q9 What is your age?

________________________________________________________________

Q10 What is your sex?

- Male
- Female
- Other

Q11 Choose one or more races that you consider yourself to be:

- White
- Black or African Americans
- Hispanic and Latino Americans
- American Indian or Alaska Native
- Asian and Asian Americans
- Native Hawaiian or Pacific Islander
- Other ________________________________________________

Q12 What is your marital status?

- Single
- Married or domestic partnership
- Widowed
- Divorced
- Separated

Q13 Over the past month, have you been mostly living alone?

- Yes
- No

Q14 What is the highest level of school you have completed or the highest degree you have received?

- Less than high school degree
- High school graduate (high school diploma or equivalent including GED)
- Some college but no degree
- Associate degree in college (2-year)
- Bachelor's degree in college (4-year)
- Master's degree
- Doctoral degree
- Professional degree (JD, MD)

Q15 Which one of the choices best describes your employment status?

- Self-employed
- Working full time for wages
- Working part time for wages
- Out of work
- Not able to work or disabled
- Retired
- Other________________________________________________

Q16 What was your total household income, before taxes, for 2019?  (Count all income from all household members who live with you.)

- Under $10,000
- $10,001 – $20,000
- $20,001 – $40,000
- $40,001 – $60,000
- $60,001 – $80,000
- $80,001 – $100,000
- $100,001 – $120,000
- More than $120,000
